# Supplementary material for: Unseen Insights: An AI-Powered Exploration of Secure Patient Messages in Ophthalmology
Source: medRxiv. 2026 Feb 5:2026.02.03.26345491. Preprint. [Version 1] doi: 10.64898/2026.02.03.26345491 (PMC12889765; doi:10.64898/2026.02.03.26345491)
Supplement: 1 [file NIHPP2026.02.03.26345491V1-supplement-1.pdf]

medRxiv preprint doi: https://doi.org/10.64898/2026.02.03.26345491; this version posted February 5, 2026. The copyright holder for this preprint (which was not certified by peer review) is the author/funder, who has granted medRxiv a license to display the preprint in perpetuity.
It is made available under a CC-BY 4.0 International license .

Supplement

Ophthalmic ICD-10 Codes	
**Amblyopia**	H53.001, H53.002, H53.003, H53.009, H53.011, H53.012, H53.013, H53.019, H53.021, H53.022, H53.023, H53.029, H53.031, H53.032, H53.033, H53.039, H53.041, H53.042, H53.043, H53.049
**Uveitis**	H20.011, H20.012, H20.013, H20.019, H20.021, H20.022, H20.023, H20.029, H20.031, H20.032, H20.033, H20.039, H20.041, H20.042, H20.043, H20.049, H20.10, H20.11, H20.12, H20.13, H20.19, H30.021, H30.022, H30.023, H30.029, H30.031, H30.032, H30.033, H30.039, H30.21, H30.22, H30.23, H30.29, H30.91, H30.92, H30.93, H30.90, H44.011, H44.012, H44.013, H44.019, H44.021, H44.022, H44.023, H44.029, H44.111, H44.112, H44.113, H44.119, H44.131, H44.132, H44.133, H44.139, H43.89, A18.54, B00.51, B02.32, M02.3, L40.50, M45.9, K52.8
**Lens**	H26.011, H26.012, H26.013, H26.019, H26.031, H26.032, H26.033, H26.039, H26.041, H26.042, H26.043, H26.049, H26.051, H26.052, H26.053, H26.059, H26.131, H26.132, H26.133, H26.139, H26.491, H26.492, H26.493, H26.499, H27.01, H27.02, H27.03, H27.00, H27.111, H27.112, H27.113, H27.119
**Blepharitis, Eyelid, Orbit, & Meibomian Gland Dysfunction**	H00.011, H00.012, H00.013, H00.014, H00.015, H00.016, H00.019H00.021, H00.022, H00.023, H00.024, H00.025, H00.026, H00.029H00.031, H00.032, H00.033, H00.034, H00.035, H00.036, H00.039, H00.11, H00.12, H00.13, H00.14, H00.15, H00.16, H00.19, H01.001, H01.002, H01.004, H01.005, H01.00A, H01.00B, H01.011, H01.012, H01.013, H01.014 H01.015, H01.016, H01.019, H01.01A, H01.01B, H01.021, H01.022, H01.024, H01.025, H01.02A, H01.02B, H02.051, H02.052, H02.053, H02.054, H02.055, H02.056, H02.059, H02.30, H02.31, H02.32, H02.33, H02.34, H02.35, H02.36, H02.411, H02.412, H02.413, H02.419, H02.421, H02.422, H02.423, H02.429, H02.431, H02.432, H02.433, H02.439, H02.521, H02.522, H02.523, H02.524, H02.525, H02.526, H02.529, H02.821, H02.822, H02.823, H02.824, H02.825, H02.826, H02.829, H02.831, H02.832, H02.833, H02.834, H02.835, H02.836, H02.839, H02.841, H02.842, H02.843, H02.844, H02.845, H02.846, H02.849, H02.881, H02.882, H02.884, H02.885, H02.88A, H02.88B, H05.221, H05.222, H05.223, H05.229, H05.231, H05.232, H05.233, H05.239, H05.241, H05.242, H05.243, H05.249, H05.251, H05.252, H05.253, H05.259, H05.261, H05.262, H05.263, H05.269, H57.811, H57.812, H57.813, H57.819, C43.111, C43.112, C43.121, C43.122, C44.1111, C44.1121, C44.1122, C44.1191, C44.1192, Q10.0, G24.5
**Refractive Errors**	H52.01, H52.02, H52.03, H52.09, H52.11, H52.12, H52.13, H52.19, H52.211, H52.212, H52.213, H52.219, H52.221, H52.222, H52.223, H52.229, Z01.01, Z01.00, Z0.0021, Z01.020, Z21.121, Z21.129
**Ocular Effects in Systemic Diseases**	E10.9, E79.60, E79.62, G70.00, G70.01, M06.0A, M06.0A, M08.00, M08.0A, M08.2A, Q68.0, Q79.61, Q79.62, Q79.69, Q82.5, Q85.01, Q85.09, Q85.8, Q87.11, Q87.42, Q90.9
**Fracture**	S02.30X-, S02.31X-, S02.32X-, S02.40A-, S02.40B-, S02.40C-, S02.40D-, S02.40E-, S02.40F-, S02.121-, S02.122-, S02.129-, S02.400-, S02.401-, S02.403-, S02.831-, S02.832-, S02.839-, S02.841-, S02.842-, S02.849-
**Lacrimal**	H04.011, H04.012, H04.013, H04.019, H04.021, H04.022, H04.023, H04.029, H04.111, H04.112, H04.113, H04.119, H04.121, H04.122, H04.123, H04.129, H04.131, H04.132, H04.133, H04.139, H04.521, H04.522, H04.523, H04.529, H04.211, H04.212, H04.213, H04.219, H04.221, H04.222, H04.223, H04.229, H04.541, H04.542, H04.543, H04.549, H04.551, H04.552, H04.553, H04.559, H04.561, H04.562, H04.563, H04.569
**Conjunctivitis**	B30.1, B30.9, H04.121, H04.122, H04.123, H04.129, H10.11, H10.12, H10.13, H11.151, H11.152, H11.153, H11.159, H10.19, H10.211, H10.212, H10.213, H10.219, H10.231, H10.232, H10.233, H10.239, H10.31, H10.32, H10.33, H10.39, H10.401, H10.402, H10.403, H10.409, H10.45, H11.811, H11.812, H11.813, H11.819, H11.821, H11.822, H11.823, H11.829, H18.831, H18.832, H18.833, H18.839, H18.891, H18.892, H18.893, H18.899, M35.00, Y77.11
**Corneal Dystrophy**	H18.501, H18.502, H18.503, H18.509, H18.511, H18.512, H18.513, H18.519, H18.521, H18.522, H18.523, H18.529, H18.531, H18.532, H18.533, H18.539, H18.541, H18.542,

medRxiv preprint doi: https://doi.org/10.64898/2026.02.03.26345491; this version posted February 5, 2026. The copyright holder for this preprint (which was not certified by peer review) is the author/funder, who has granted medRxiv a license to display the preprint in perpetuity.
It is made available under a CC-BY 4.0 International license .

	H18.543, H18.549, H18.551, H18.552, H18.553, H18.559, H18.591, H18.592, H18.593, H18.599
**Corneal Edema/Opacity/ Degeneration**	H18.11, H18.12, H18.13, H18.19, H18.211, H18.212, H18.213, H18.219, H18.221, H18.222, H18.223, H18.229, H18.231, H18.232, H18.233, H18.239, H18.331, H18.332, H18.333, H18.339, H18.411, H18.412, H18.413, H18.419, H18.421, H18.422, H18.423, H18.429, H18.441, H18.442, H18.443, H18.449, H18.451, H18.452, H18.453, H18.459, H18.461, H18.462, H18.463, H18.469, H18.611, H18.612, H18.613, H18.619, H18.621, H18.622, H18.623, H18.629, H18.711, H18.712, H18.713, H18.719, H18.731, H18.732, H18.733, H18.739, H17.11, H17.12, H17.13, H17.19, H17.811, H17.812, H17.813, H17.819, H17.821, H17.822, H17.823, H17.829, H17.89
**Corneal Transplant**	S05.01X-, S05.02X-, S05.21X-, S05.22X-, S05.31X-, S05.32X, T86.8401, T86.8402, T86.8403, T86.8411, T86.8412, T86.8413, T86.8421, T86.8422, T86.8423, T86.8481, T86.8482, T86.8483, T86.8491, T86.8492, T86.8493, Z94.7
**Esotropia**	H50.00, H50.011, H50.012, H50.021, H50.022, H50.031, H50.032, H50.041, H50.042, H50.05, H50.06, H50.07, H50.08, H50.312, H50.311, H50.32, H50.43
**Endophthalmitis**	H44.001, H44.002, H44.003, H44.009, H44.011, H44.012, H44.013, H44.019, H44.111, H44.112, H44.113, H44.119, H44.131, H44.132, H44.133, H44.139, H44.19
**Lens**	H26.011, H26.012, H26.013, H26.019, H26.031, H26.032, H26.033, H26.039, H26.041, H26.042, H26.043, H26.049, H26.051, H26.052, H26.053, H26.059, H26.111, H26.112, H26.113, H26.119, H26.131, H26.132, H26.133, H26.139, H26.491, H26.492, H26.493, H26.499, H27.00, H27.01, H27.02, H27.03, H27.111, H27.112, H27.113, H27.119
**Neuro-ophthalmic/ Optic Nerve Disorders**	H46.00, H46.01, H46.011, H46.012, H46.013, H46.019, H46.02, H46.03, H46.10, H46.11, H46.12, H46.13, H46.2, H46.3, H46.8, H46.9, H47.011, H47.012, H47.013, H47.019, H47.091, H47.092, H47.093, H47.099, H47.10, H47.11, H47.20, H47.22, H47.41, H47.42, H47.43, H47.49
**Ectropion**	H02.111, H02.112, H02.114, H02.115, H02.121, H02.122, H02.124, H02.125, H02.131, H02.132, H02.134, H02.135, H02.141, H02.142, H02.144, H02.145, H02.151, H02.152, H02.154, H02.155
**Amblyopia**	H53.011, H53.012, H53.013, H53.019, H53.021, H53.022, H53.023, H53.029, H53.031, H53.032, H53.033, H53.039, H53.041, H53.042, H53.043, H53.049, H53.001, H53.002, H53.003, H53.009
**Hereditary Retinal Dystrophies**	H35.51, H35.52, H35.53, H35.54
**Iridocyclitis**	H20.011, H20.012, H20.013, H20.019, H20.10, H20.11, H20.12, H20.13, H20.20, H20.21, H20.22, H20.23, H20.029, H20.021, H20.022, H20.023, H20.039, H20.031, H20.032, H20.033, H20.049, H20.041, H20.042, H20.043
**Entropion**	H02.011, H02.012, H02.014, H02.015, H02.021, H02.022, H02.024, H02.025, H02.031, H02.032, H02.034, H02.035, H02.041, H02.042, H02044, H02.045, H02.051, H02.052, H02.054, H02.055
**Exotropia**	H50.10, H50.15, H50.16, H50.17, H50.18, H50.111, H50.121, H50.131, H50.141, H50.112, H50.122, H50.132, H50.142, H50.331, H50.332, H50.34
**Neurologic disorders**	G24.5, G35, G43.B0, G43.B1, G43.E01, G43.E09, G43.E11, G43.E19, G45.3, G45.9, G51.31, G51.32, G51.33, G51.39, G70.00, G70.01, G90.2, G93.2, G93.5
**Choroidal Disorders**	D31.30, D31.301, D31.302, D31.303, D31.309, D31.31, D31.32, D31.321, D31.322, D31.323, D31.329, H30.90, H30.91, H30.92, H30.93, H31.011, H31.012, G31.013, G31.019, H31.021, H31.022, H31.023, H31.029, H31.411, H31.412, G31.413, G31.419, H31.421, H31.422, H31.423, H31.429, H59.811, H59.812, H59.813, H59.819
**Viral/Keratitis**	B00.51, B00.52, B00.53, B00.59, B02.31, B02.32, B02.33, B02.34, B02.39, H16.011, H16.012, H16.013, H16.019, H16.021, H16.022, H16.023, H16.029, H16.031, H16.032, H16.033, H16.039, H16.041, H16.042, H16.043, H16.049, H16.051, H16.052, H16.053, H16.059, H16.061, H16.062, H16.063, H16.069, H16.071, H16.072, H16.073, H16.079, H16.121, H16.122, H16.123, H16.129, H16.131, H16.132, H16.133, H16.139, H16.141, H16.142, H16.143, H16.149, H16.211, H16.212, H16.213, H16.219, H16.221, H16.222, H16.223, H16.229, H16.231, H16.232, H16.233, H16.239, H16.251, H16.252, H16.253, H16.259,

medRxiv preprint doi: https://doi.org/10.64898/2026.02.03.26345491; this version posted February 5, 2026. The copyright holder for this preprint (which was not certified by peer review) is the author/funder, who has granted medRxiv a license to display the preprint in perpetuity.
It is made available under a CC-BY 4.0 International license .

	H16.291, H16.292, H16.293, H16.299, H16.311, H16.312, H16.313, H16.319, H16.321, H16.322, H16.323, H16.329, H16.431, H16.432, H16.433, H16.439, M35.01
**Foreign body/injury/ laceration/mass**	D18.01, D18.09, D31.60, D31.61, D31.62, T26.11XA, T26.11XD, T26.11XS
**Retinopathy of Prematurity**	H35.111, H35.112, H35.113, H35.119, H35.121, H35.122, H35.123, H35.129, H35.131, H35.132, H35.133, H35.139, H35.141, H35.142, H35.143, H35.149, H35.151, H35.152, H35.153, H35.159, H35.161, H35.162, H35.163, H35.169, H35.101, H35.102, H35.103, H35.109
**Type I Diabetic Retinopathy**	E10.3211, E10.3212, E10.3213, E10.3219, E10.3311, E10.3312, E10.3313, E10.3319, E10.3411, E10.3412, E10.3413, E10.3419, E10.3511, E10.3512, E10.3513, E10.3519, E10.3521, E10.3522, E10.3523, E10.3529, E10.3541, E10.3542, E10.3543, E10.3549, E10.37X1, E10.37X2, E10.37X3, E10.37X
	E10.3291, E10.3292, E10.3293, E10.3299, E10.3391, E10.3392, E10.3393, E10.3399, E10.3491, E10.3492, E10.3493, E10.3499, E10.3531, E10.3532, E10.3533, E10.3539, E10.3551, E10.3552, E10.3553, E10.3559, E10.3591, E10.3592, E10.3593, E10.3599, E10.9
**Type II Diabetic Retinopathy**	H35.3190, H35.3191, H35.3192, H35.3193, H35.3194, E11.3211, E11.3212, E11.3213, E11.3219, E11.3291, E11.3292, E11.3293, E11.3299, E11.3311, E11.3312, E11.3313, E11.3319, E11.3391, E11.3392, E11.3393, E11.3399, E11.3411, E11.3412, E11.3413, E11.3419, E11.3491, E11.3492, E11.3493, E11.3499, E11.3511, E11.3512, E11.3513, E11.3519, E11.3521, E11.3522, E11.3523, E11.3529, E11.3531, E11.3532, E11.3533, E11.3539, E11.3541, E11.3542, E11.3543, E11.3549, E11.3551, E11.3552, E11.3553, E11.3559, E11.3591, E11.3592, E11.3593, E11.3599
	H35.341, H35.342, H35.351. H35.352, H35.353, H35.359, H35.361, H35.362, H35.363, H35.369, H35.371, H35.372, H35.373, H35.379, H35.433, H35.439, H35.711, H35.712, H35.713, H35.719, H43.821, H43.822, H43.823, H43.829, H35.3110, H35.3111, H35.3112, H35.3113, H35.3114, H35.3120, H35.3121, H35.3122, H35.3123, H35.3124, H35.3130, H35.3131, H35.3132, H35.3133, H35.3134, H35.3210, H35.3211, H35.3212, H35.3213, H35.3220, H35.3221, H35.3222, H35.3223, H35.3230, H35.3231, H35.3232, H35.3233, H35.3290, H35.3291, H35.3292, H35.3293, H59.031, H59.032, H59.033, H59.039
**Lagophthalmos**	H02.201, H02.202, H02.204, H02.205, H02.20A, H02.20B, H02.20C, H02.211, H02.212, H02.214, H02.215, H02.21A, H02.21B, H02.21C, H02.221, H02.222, H02.224, H02.225, H02.22A, H02.22B, H02.22C, H02.231, H02.232, H02.234, H02.235, H02.23A, H02.23B, H02.23C, H02.531, H02.532, H02.534, H02.535
**Glaucoma**	H10.011, H10.012, H10.013, H40.001, H40.002, H40.003, H40.011, H40.012, H40.013, H40.021, H40.022, H40.023, H40.031, H40.032, H40.033, H40.041, H40.042, H40.043, H40.051, H40.052, H40.053, H40.061, H40.062, H40.063, H40.10X2, H40.20X1, H40.111-, H40.112-, H40.113-, H40.121-, H40.122-, H40.123-, H40.131-, H40.132-, H40.133-, H40.141-, H40.142-, H40.143-, H40.151, H40.152, H40.153, H40.211-, H40.212-, H40.213-, H40.221-, H40.222-, H40.223-, H40.231-, H40.232-, H40.233-, H40.241-, H40.242-, H40.243-, H40.31X-, H40.32X-, H40.33X-, H40.31X3, H40.41X-, H40.42X-, H40.43X-, H40.43X2, H40.51X-, H40.52X-, H40.53X-, H40.61X-, H40.62X-, H40.63X-, H40.811, H40.812, H40.813, H40.821, H40.822, H40.823, H40.831, H40.832, H40.833, H44.411, H44.412, H44.413, H44.421, H44.422, H44.423, H44.511, H44.512, H44.513, P15.3, Q15.0, T49.5X5, T49.5X6, T50.2X5, , T81.31X, T81.83X, T85.390, T85.391, T85.398
**Myopic Degeneration**	H44.2A1, H44.2A2, H44.2A3, H44.2A9, H44.2B1, H44.2B2, H44.2B3, H44.2B9, H44.2C1, H44.2C2, H44.2C3, H44.2C9, H44.2D1, H44.2D2, H44.2D3, H44.2D9, H44.2E1, H44.2E2, H44.2E3, H44.2E9, H44.21, H44.22, H44.23
**Vitreous Body Degeneration**	H43.10, H43.11, H43.12, H43.13, H43.20, H43.21, H43.22, H43.23, H43.391, H43.392, H43.393, H43.399, H43.811, H43.812, H43.813, H43.819, H43.821, H43.822, H43.823, H43.829, H43.89
**Panuveitis**	H44.00-, H44.11-, H44.12-, H44.13-, H44.02-, H20.03-, H20.04-
**Palsies**	H49.00, H49.01, H49.02, H49.03, H49.10, H49.11, H49.12, H49.13, H49.20, H49.21, H49.22, H49.23, H49.30, H49.31, H49.32, H49.33, H49.40, H49.41, H49.42, H49.43, H49.881, H49.882, H49.883, H49.889, H49.9
**Pterygium**	H11.021, H11.022, H11.023, H11.029, H11.031, H11.032, H11.033, H11.039, H11.041,

medRxiv preprint doi: https://doi.org/10.64898/2026.02.03.26345491; this version posted February 5, 2026. The copyright holder for this preprint (which was not certified by peer review) is the author/funder, who has granted medRxiv a license to display the preprint in perpetuity.
It is made available under a CC-BY 4.0 International license .

	H11.042, H11.043, H11.049, H11.051, H11.052, H11.053, H11.059, H11.061, H11.062, H11.063, H11.069
**Nystagmus**	H55.00, H55.01, H55.02, H55.03, H55.04, H55.09, H55.81, H55.89
**Strabismus**	H44.22A1, H44.22A2, H44.22A3, H44.22A9, H44.22B1, H44.22B2, H44.22B3, H44.22B9, H44.22C1, H44.22C2, H44.22C3, H44.22C9, H44.22D1, H44.22D2 H44.22D3, H44.22D9, H44.22E1, H44.22E2, H44.22E3, H44.22E9, H50.21, H50.22, H50.50, H50.51, H50.52, H50.53, H50.54, H50.55, H50.60, H50.611, H50.612, H50.69, H50.811, H50.812, H50.89, H50.9, Q10.3
**Vertigo**	H02.411, H02.412, H02.413, H02.419, H02.421, H02.422, H02.423, H02.429, H02.431, H02.432, H02.433, H02.439, Q10.0
**Visual Disturbances**	H53.021, H53.022, H53.023, H53.029, H53.031, H53.032, H53.033, H53.039, H53.19, H53.2, H53.40, H53.47, H53.421, H53.422, H53.423, H53.429, H53.461, H53.462, H53.469, H53.52
**Vision Rehabilitation**	H54.0X33, H54.0X34, H54.0X35, H54.0X43, H54.0X44, H54.0X45, H54.0X53, H54.0X54, H54.0X55, H54.1131, H54.1132, H54.1131, H54.1141, H54.1142, H54.1151, H54.1152, H54.1213, H54.1214, H54.1215, H54.1223, H54.1224, H54.1225, H54.2X11, H54.2X21, H54.2X12, H54.2X22, H54.413A, H54.414A, H54.415A,H 54.42A3, H54.42A4, H54.42A5, H54.511A, H54.512A, H54.52A1, H54.52A2
**Vitreous Body Degeneration**	H43.11, H43.12, H43.13, H43.10, H43.21, H43.22, H43.23, H43.20, H43.391, H43.392, H43.393, H43.399, H43.811, H43.812, H43.813, H43.819, H43.821, H43.822, H43.823, H43.829
**Peripheral Retinal Degeneration**	H35.411, H35.412, H35.413, H35.419, H35.421, H35.422, H35.423, H35.429, H35.431, H35.432, H35.433, H35.439, H35.441, H35.442, H35.443, H35.449, H35.461, H35.462, H35.463, H35.469
**Retinal Detachments**	H33.011, H33.012, H33.013, H33.019, H33.101, H33.102, H33.103, H33.109, H33.191, H33.192, H33.193, H33.199, H33.031, H33.032, H33.033, H33.039, H33.041, H33.042, H33.043, H33.049, H33.051, H33.052, H33.053, H33.059, H33.21, H33.22, H33.23, H33.20, H33.41, H33.42, H33.43, H33.49, H33.8
**Retinal Tears**	H33.301, H33.302, H33.303, H33.309, H33.311, H33.312, H33.313, H33.319, H33.321, H33.322, H33.323, H33.329, H33.331, H33.332, H33.333, H33.33
**Retinal Vascular Occlusion**	H34.10, H34.11, H34.12, H34.13, H34.8110, H34.8120, H34.8130 H34.8190, H34.8111, H34.8121, H34.8131, H34.8191, H34.8112, H34.8122, H34.8132, H34.8192, H34.8310, H34.8320, H34.8330, H34.8390, H34.8311, H34.8321, H34.8331, H34.8391, H34.8312, H34.8322, H34.8332, H34.8392
**Separation of Retinal Layers**	H35.711, H35.712, H35.713, H35.719, H35.721, H35.722, H35.723, H35.729, H35.731, H35.732, H35.733, H35.739
**Other Retinal Disorders**	H35.021, H35.022, H35.023, H35.029, H35.031, H35.032, H35.033, H35.039, H35.041, H35.042, H35.043, H35.049, H35.051, H35.052, H35.053, H35.059, H35.20, H35.21, H35.22, H35.23, H35.60, H35.61, H35.62, H35.63, H35.81, H35.82
**Miscellaneous**	C69.20, C69.21, C69.22, D31.60, D31.61, D31.62, D32.0, D35.2, E05.00, E05.01, F08.71, F44.679, G24.5, G43.B0, G43.B1, G43.101, G43.109, G43.111, G43.119, G43.801, G43.809, G43.811, G43.819, G45.3, G45.9, G51.31, G51.32, G51.33, G51.39, G80.9, H02.89, H05.111, H05.112, H05.113, H05.119, H30.011, H30.012, H30.012, H30.019, H34.211, H34.212, H34.213, H34.219, H44.521, H44.522, H44.523, H44.529, H47.611, H47.612, H49.41, H49.42, H49.43, H49.40, H51.11, H51.21, H51.22, H51.23, H51.20, H51.8, H53.131, H53.132, H53.133, H53.1139, H53.121, H53.122, H53.123, H53.129, H53.19, H53.2, H53.8, H54.7, H57.051, H57.052, H57.053, H57.059, H57.11, H57.12, H57.13, H57.10, H57.89, H57.02, H57.9, H59.021, H59.022, H59.023, H59.029, I63.9, L57.0, L82.1, L93.0, M06.9, P04.40, Q07.8, Q13.1, R51.0, R51.9, R48.3, R70.0, R73.03, R73.09, S00.11X-, S00.12X-, S05.01X-, S05.00X-, S05.02X-, T15.01X-, T15.02X-, T15.10X-, T15.11X-, T15.12X-, T15.81X-, T15.82X-, T26.00X-, T26.01X-, T26.02X-, Z05.8, Z79.899
**Age-Related Macular Degeneration**	H35.3111, H35.3112, H35.3113, H35.3121, H35.3122, H35.3123, H35.3131, H35.3132, H35.3133, H35.3211, H35.3212, H35.3213, H35.3221, H35.3222, H35.3223, H35.3231, H35.3232, H35.3233

eTable 1. Ophthalmic ICD-10 diagnostic codes used to identify patients in this study

medRxiv preprint doi: https://doi.org/10.64898/2026.02.03.26345491; this version posted February 5, 2026. The copyright holder for this preprint (which was not certified by peer review) is the author/funder, who has granted medRxiv a license to display the preprint in perpetuity.
It is made available under a CC-BY 4.0 International license .

**Predfined Message Topics**	**N**	**%**
Scheduling/canceling	4166	13.7
Vision	2975	9.8
Pharmacy/medication	1352	4.4
Glaucoma	1241	4.1
Tumor	1074	3.5
Surgery	1064	3.5
Complications/other conditions*	774	2.5
Insurance	581	1.9
Swell/infection	525	1.7
Cornea	311	1.0
Disability	221	0.7
**Total pre-defined messages**	**14284**	**50.3**
Other undefined messages	16106	49.7
**Total All Messages**	**30390**	**100.0**

eTable 2. Messages categorization based on pre-definition. *Complications/other conditions: other conditions or referrals not covered by the list in the current table, including pneumonia, myasthenia gravis, vertigo, or thyroid issues

medRxiv preprint doi: https://doi.org/10.64898/2026.02.03.26345491; this version posted February 5, 2026. The copyright holder for this preprint (which was not certified by peer review) is the author/funder, who has granted medRxiv a license to display the preprint in perpetuity.
It is made available under a CC-BY 4.0 International license .

**Administrative issues**	**Scheduling**	Patients coordinate ophthalmology visits—scheduling, rescheduling, cancellations, and referrals for in-person or video consults—and need clear communication with schedulers, technicians, and providers for timely care.
	**Pharmacy refill**	Patients struggle to refill prednisone/prednisolone at retail pharmacies, handling authorizations, pickups, and bottle exchanges while seeking relief from persistent swelling.
	**Insurance**	Patients struggle with insurance authorizations—denials, Medicare navigation, and appeals—and billing processes, causing financial stress and delaying essential ophthalmic care.
	**Disability**	Patients with vision-limiting eye disease apply for SSDI and FMLA, burdened by complex paperwork—completing, submitting, and mailing forms—that delays their benefit access.
**Clinical Issues**	**Vision**	Patients seek updated glasses or contact lens prescriptions for blurred, tilted, or floating vision, undergo stereotests and refraction, and have retinopathy, macular changes, or keratoconus, prompting LASIK discussion.
	**Glaucoma**	Glaucoma and ocular hypertension patients use topical/systemic pressure□lowering agents (timolol, dorzolamide, latanoprost, Lumigan, Zioptan, Diamox), request renewals, schedule IOP checks, and manage discomfort with OTC analgesics.
	**Surgery**	Patients undergoing ocular procedures (Botox, strabismus surgery, blepharoplasty) focus on perioperative care—anesthesia, incision management, steroids, antibiotics, antihistamines—and address swelling, bleeding, infection, itching, and tear support.
	**Tumor**	Patients undergo imaging (with/without contrast), blood tests, biopsies, and genetic analysis to diagnose ocular/orbital lesions, then receive targeted treatments like laser procedures.
	**Complications**	Patients with myasthenia gravis (Mestinon), thyroid orbitopathy (Tepezza), or autoimmune disease (Plaquenil) report vision disturbances requiring neurologic, rheumatologic, and endocrine coordination, invasive procedures, and immunosuppression management.
	**Swelling**	Patients experience eyelid/eyelash swelling (styes/infections) and conjunctivitis managed with drops; some seek surgical eyelid/brow incisions for persistent lesions and schedule acute eye-care appointments.
	**Cornea**	Patients with cataracts schedule lens-replacement to treat vision loss, coordinating preoperative assessments, surgery logistics, and postoperative care for restored vision.

eTable 3. Ophthalmic message topics classified by administrative versus clinical issues

medRxiv preprint doi: https://doi.org/10.64898/2026.02.03.26345491; this version posted February 5, 2026. The copyright holder for this preprint (which was not certified by peer review) is the author/funder, who has granted medRxiv a license to display the preprint in perpetuity.
It is made available under a CC-BY 4.0 International license .

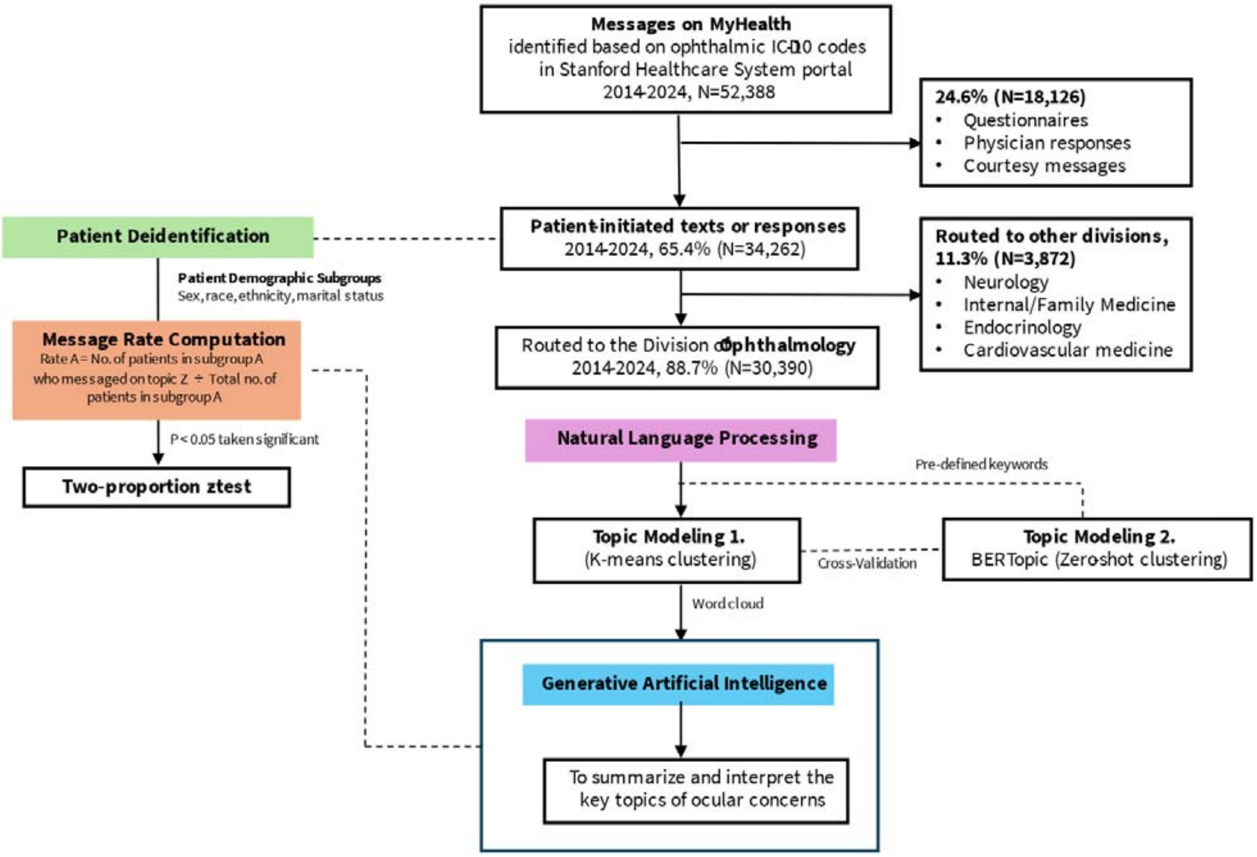

382

383 **eFigure 1.** Workflow of patient message data extraction, processing, and analysis

384
